# Carroll County Medical Society

**Published:** 1869-04

**Authors:** Jno. L. Hostetter, J. Haller

**Affiliations:** President; Secretary


### CARROLL COUNTY MEDICAL SOCIETY.

The second regular meeting of this society was held in Lanark, on the 23d of February, ultimo.

Dr. John L. Hostetter, President elect, presiding.

Minutes of previous meeting were read and approved.

Dr. N. Stepenson, of Thomson, read a paper on the nature of the action of chloroform as an anæsthetic agent.

This was a very able production, and showed that the Doctor has been a very close observer of the action of this article. He advanced some very correct ideas as to its peculiar action in some cases, and gave some very excellent advice as to the mode of administering this agent.

Dr. J. Haller, of Lanark, read a paper on the action of gelseminum sempervirens, but modesty will not allow any comment upon our own paper.

Some of the members having been requested to read papers asked for further time.

There was considerable discussion on some of the ideas set forth in the papers read, which passed off very agreeably to all. This is what we want, *viz.:* free expression of views, and reports of our experience in these matters. By so doing we will be enabled to combat disease more successfully, and make the practice of our noble profession more pleasant.

Dr. Hostetter not having a paper prepared on medical ethics offered a verbal report, in a few well-timed words, to wit:—"Labor diligently and unitedly for the good of suffering humanity." "In all things whatsoever ye would that men should do to you, do ye even so to them."

The following were requested to read papers at our next meeting (and also those who did not read at this meeting are requested to be prepared at our next):—

On obstetrics, Dr. Jno. L. Hostetter, of Mount Carroll.

On the etiology and nature of "shock," Dr. N. Stephenson, of Thomson.

On motion of Dr. J. Haller, it was decided to hold the next meeting of this society in Mount Carroll, on the second Tuesday of June next, at 10 o'clock A.M. We hope to see a good attendance at that time. All members of the press and clergymen favorable to a *rational* system of medicine are invited to meet with us.

JNO. L. HOSTETTER, *President.*

J. HALLER, M.D., *Secretary.*

---

